# The Pet Factor - Companion Animals as a Conduit for Getting to Know People, Friendship Formation and Social Support

**DOI:** 10.1371/journal.pone.0122085

**Published:** 2015-04-29

**Authors:** Lisa Wood, Karen Martin, Hayley Christian, Andrea Nathan, Claire Lauritsen, Steve Houghton, Ichiro Kawachi, Sandra McCune

**Affiliations:** 1 School of Population Health, The University of Western Australia, Crawley, Western Australia, Australia; 2 Centre for Built Environment and Health, The University of Western Australia, Crawley, Western Australia, Australia, and Telethon Kids Institute, Perth, Western Australia, Australia; 3 School of Public Health and Social Work, Queensland University of Technology, Kelvin Grove, Queensland, Australia; 4 Graduate School of Education, The University of Western Australia, Crawley, Western Australia, Australia; 5 Department of Social and Behavioral Sciences, Harvard School of Public Health, Harvard University, Cambridge, Massachusetts, United States of America; 6 WALTHAM Centre for Pet Nutrition, Leicestershire, United Kingdom; University of Utah, UNITED STATES

## Abstract

**Background:**

While companion animals have been previously identified as a direct source of companionship and support to their owners, their role as a catalyst for friendship formation or social support networks among humans has received little attention. This study investigated the indirect role of pets as facilitators for three dimensions of social relatedness; getting to know people, friendship formation and social support networks.

**Methods:**

A telephone survey of randomly selected residents in four cities, one in Australia (Perth; *n* = 704) and three in the U.S. (San Diego, *n* = 690; Portland, *n* = 634; Nashville, *n* = 664) was conducted. All participants were asked about getting to know people within their neighborhood. Pet owners were asked additional questions about the type/s of pet/s they owned, whether they had formed friendships as a result of their pet, and if they had received any of four different types of social support from the people they met through their pet.

**Results:**

Pet owners were significantly more likely to get to know people in their neighborhood than non-pet owners (OR 1.61; 95%CI: 1.30, 1.99). When analyzed by site, this relationship was significant for Perth, San Diego and Nashville. Among pet owners, dog owners in the three U.S. cities (but not Perth) were significantly more likely than owners of other types of pets to regard people whom they met through their pet as a friend (OR 2.59; 95%CI: 1.94, 3.46). Around 40% of pet owners reported receiving one or more types of social support (i.e. emotional, informational, appraisal, instrumental) via people they met through their pet.

**Conclusion:**

This research suggests companion animals can be a catalyst for several dimensions of human social relationships in neighborhood settings, ranging from incidental social interaction and getting to know people, through to formation of new friendships. For many pet owners, their pets also facilitated relationships from which they derived tangible forms of social support, both of a practical and emotionally supportive nature. Given growing evidence for social isolation as a risk factor for mental health, and, conversely, friendships and social support as protective factors for individual and community well-being, pets may be an important factor in developing healthy neighborhoods.

## Introduction

Pets play an important role in the lives of many people throughout the world, and there is a growing body of research indicating a positive relationship between pet ownership and human health [1–5]. With more than 60% of households in the U.S. and Australia owning one or more pets [6,7], the benefits to individuals of human-animal interactions (HAI) to health have been widely explored. Findings have revealed a number of therapeutic, physiological, psychological and psychosocial benefits to pet owners [8] including decreased blood pressure [9,10], reduced risk of heart attacks [11], improved survival rates [9,12,13], increased physical activity [14], increased sensory stimulation, emotional support and sense of physical and psychological well-being [15], as well as psychological resilience at times of adversity [16,17].

In the literature more broadly, there is now compelling empirical evidence for the importance of social relationships and social support for both physical and mental health and wellbeing. Indeed, a meta-analysis of 148 studies placed the influence of social relationships on mortality risk on a par with well-established risk factors such as smoking and alcohol consumption [5], concluding that people had a 50 percent higher survival rate if they belonged to a wider social group. Conversely, social isolation and loneliness have been identified as risk factors for poor health [18].

How then can socially supportive networks be enhanced in a modern fast-paced world that has been observed by some sociologists to precipitate disconnectedness from the people amongst whom we live [19]? Although there are anecdotal and qualitative research accounts about pets as a ‘social lubricant’ [20,21], this has rarely been investigated empirically. Research into the role of animals in facilitating social interaction among human beings has lagged behind the proliferation of research into other health or therapeutic benefits associated with companion animals [22]. Of the studies undertaken to date, the focus has primarily been on dogs in their capacity as social ‘ice-breakers’ [20], with a number reporting dogs were often a conversation trigger between strangers or casual acquaintances [23–25]. Several experimental studies have reported dog walkers are more likely to experience social contact and conversation than solitary walkers [3,23,26]. This conversational ice-breaker role is not limited to dogs, with other experimental studies finding small animals such as turtles and rabbits also precipitating conversation between strangers in a park setting [27]. One rationale put forth by McNicholas and Collis [26] is that the presence of an animal provides people with a neutral and safe conversation starter.

Other evidence suggests pets can precipitate more than just incidental contact or casual conversations with strangers. For instance, in a previously published study undertaken in Perth, Western Australia, 40.5% of pet owners reported getting to know people in their suburb as a result of their pet [8]. Knowing people within the local community can be an important antidote to isolation and social disconnectedness [28], regardless of whether or not it deepens into friendships [29].

Further along the social relatedness continuum, the role of pets in facilitating new friendships has been observed in qualitative studies in Australia [30] and the UK [31] but has been less explored empirically.

While companion animals have been recognised as a source of social support for their owners in the HAI literature [32], pets may also precipitate human-human interactions that lead to supportive networks [33]. Qualitative data from a previous Perth study suggested relationships facilitated through pets can evolve into a source of social support for some pet owners [21]. By contrast, a UK study by Collis et al. [34] contended that casual interactions facilitated by dogs did not necessarily enhance social networks or social support. These differing findings highlight the importance of distinguishing between the different types of ‘social lubrication’ that might be precipitated by pets; ranging from incidental greetings exchanged with a stranger, through to getting to know people by face or name, through to people being considered an acquaintance or even a friend. Social support can differ again, and, as articulated by Collis et al. [34], may not necessarily transpire from casual interactions catalyzed by companion animals.

**Fig 1:**
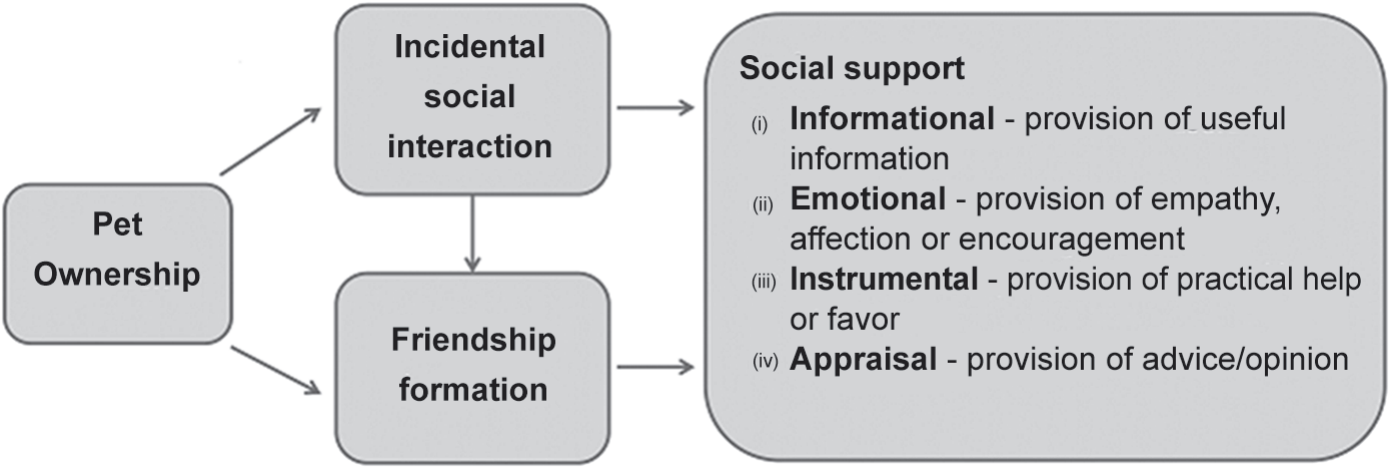
Types of social relatedness facilitated by pets.

Fig 1 below presents a conceptual diagram of the main types of pet-facilitated social relatedness identified from the literature considered as part of this study. The inclusion of social support as a potential byproduct of pet ownership is a particularly novel proposition within the domain of HAI research, where social support has been more typically viewed as something owners might derive directly from their companion animal [32], rather than through people met via their pet.

This mixed methods study collected survey data from four cities (three in the U.S., one in Australia) and was underpinned by four research aims. These aims were to:

- investigate the extent to which pets are identified (unprompted) as one of the ways people got to know other people in their neighborhood;
- explore whether pet ownership facilitates the formation of new friendships (beyond acquaintances);
- investigate whether tangible forms of social support are derived from relationships facilitated through pet ownership; and further, to disaggregate the types of social support pet owners receive from people met through their pet;
- to assess (for the above aims), whether the likelihood of pets facilitating getting to know people, friendships or social support is greater among dog owners, compared with owners of other types of pets.

Differences between cities were also examined for the above aims. Responses to an open-ended question provided additional qualitative data on how people got to know others through their pet.

## Methods

### Study design and sample

This study was originally designed to be undertaken in one city from each of the two countries (Perth, Australia and San Diego, U.S.) with comparable population demographics, climate, coastal geography and similar patterns of residential density, suburbanism and predominant housing type. This was then expanded to select two other U.S. cities (Portland in the north-west, and Nashville in the south-east) that met similar criteria for low-medium residential density (<4390/sq. mile) [35] and moderate climate (mean annual temperature ranges between highs of 63–75 degrees °F, and lows of 45–60°F) [36]. U.S. cities with extremely cold weather were excluded from consideration as weather has the potential to influence people’s propensity to be outdoors in their neighborhood, and may impede dog walking for those who own dogs. We were also conscious that patterns of pet ownership and the nature of ‘neighborhood’ can be influenced by city characteristics such as housing type (e.g. single house versus apartment), density and geographies of residential distribution (e.g. highly concentrated inner city versus outer city or suburbs). Therefore, prior to the final selection of participating cities, population census data from a narrowed set of possible U.S. cities [37] was reviewed and compared to Perth population census data [38] including the proportion of residents living in houses versus townhouses/units or apartments/condominiums. As Perth has limited inner city apartment living, U.S. cities with a high concentration were excluded as high-rise inner city living has implications for pet ownership [39] that may have confounded the study.

The study employed a cross-sectional design entailing telephone surveying of a randomized population sample from each of the four cities during their respective autumn/early winter months. In Perth, Australia this fell between April and June 2012 and for the U.S. cities this fell between September and December 2012.

As this study was part of a larger study on social capital [40], the sample size of 630 respondents per city was calculated *a priori* to provide the study with a minimum of 80% power to detect a two-unit difference in mean social capital scale scores and between pet and non-pet owners; and, for pet owners, to be able to detect a minimum two-unit difference on social capital scores between dog and non-dog owners. A two-sample t-test for Mean Difference was used. The calculations were based on our previously published study [8] and used the most recently available pet ownership data for Australia [41] and the U.S. [42].

The study was granted ethics approval by the Human Research Ethics Committee of The University of Western Australia. Being a telephone survey, all potential participants were read the scripted participant information, and verbal consent was obtained prior to the survey commencing.

The final sample comprised 2692 respondents (San Diego *n* = 690; Nashville *n* = 664; Portland *n* = 634; Perth *n* = 704).

### Data collection

The survey was conducted using Computer Assisted Telephone Interviewing (CATI). In both countries, the survey was undertaken by an independent research agency with expertise in telephone based population surveys. Protocols were developed by the research team to ensure standardization of administration and data collection methods in all four cities. Quota specifications were used to ensure the sample contained a cross-section of sex, age group, and neighborhood socio-economic status which was representative of the wider population. To be eligible, participants had to be aged ≥18 years and to have lived in their neighborhood for at least one year. An overall response rate of 47.34% was achieved.

### Measures

The survey incorporated or adapted items from previous studies where possible, with modifications and new items informed by the literature review. Item wording, source and reliability are described below.

#### Getting to know people in neighborhood

All respondents (pet and non-pet owners) were asked, *“Have you got to know people in this neighborhood that you didn’t know before you lived here*?*”* A previously published paper reported the good-excellent test/re-test reliability of this item, with a kappa value within the 0.6–0.8 range [8], and percent agreement of 89% [43]. Those who answered “yes” were then asked *“How have you got to know these people*?*”* The telephone interviewers were trained to code the unprompted responses into the following 12 categories; being neighbors, children’s school, children (other than school), sport, clubs, church, pets, walking my/a dog, met in street or park, local shops, community event and through other friends. Those who gave a response that did not fit one of these categories were coded as ‘other’ and their responses documented verbatim.

#### Pet ownership

All respondents were asked “*Do you own a pet*?*”* followed by *“How many*, *if any*, *of the following pets do you have*?*”* and categorized into; dog, cat, bird, fish and other. These items were originally adapted from McHarg et al. [44] and the reliability established and reported by Wood et al. [8]. Respondents who reported owning a dog were also asked if they walked their dog using an item with previously demonstrated reliability [45]. This item was included to enable us to empirically test a hypothesis derived from the literature regarding dogs and dog walking as significant precipitants of meeting people in the neighborhood.

#### Getting to know people in the neighborhood through pets

Pet owners were asked “*Have you got to know people in your neighborhood as a result of your pet*? *(for example*, *through walking your pet or talking to your neighbors about your pet)”* this item was also adapted originally from McHarg et al. [44] and used in Wood et al. [8].

Respondents who reported owning a pet were asked if they had got to know people in their neighborhood as a result of their pet, and then, if they answered “yes”, they were asked “C*an you tell me a bit more about how you got to know people through your pet*?” These open-ended responses were recorded verbatim.

#### Friendship formation facilitated through pets

Pet owners who indicated they had met people in their neighborhood as a result of their pet, were also asked *“Do you regard any of the people you have met through your pet as a friend (more than just an acquaintance)*?*” (yes/no)*. This item was developed specifically for this study.

#### Social support received by pet owners from people met through their pet/s

Pet owners were asked whether they had received different types of social support from people they met through their pet, with the items discriminating between the four major types of social support described by House [46] and advocated in the research of Israel [47] and Young [48]. Based on House and Israel’s definitions and examples of the four types of social support, pet owners were asked, *“Have you met anyone through your pet who you could*:

- *talk with about something that was worrying you such as a work or family issue (emotional support);*
- *Ask for information such as, if they could recommend a tradesperson or restaurant (informational support);*
- *ask for advice (appraisal support);*
- *ask to borrow something (such as a book or tool), or ask a favor (such as collect mail) or ask for practical help such as getting a ride (instrumental support)?”*

Demographic data collected from all respondents included home ownership, dependent children, employment, education, income, age, ethnicity and gender (see S1 Appendix for sample descriptives). All respondents were also asked whether they owned a pet, and if so, what type. The respondent profile (including pet ownership rates) for each city was reasonably reflective of the demography as reported in each of the most recent U.S. and Australian National Censuses [49,50].

### Analysis

For the purposes of comparative analysis, respondents were categorized by pet ownership (pet owner versus non-pet owner). For the questions posed to pet owners only, respondents were categorized into dog owners and other pet owners. Within the dog owners group, a distinction was made between those who reported walking their dog and those who did not (dog walkers versus non-dog walkers).

All quantitative analyses were conducted using SPSS Statistics 21. Binary logistic regression was used to examine the relationship between pet owners and the categorical variables (friendship formation, pet-specific friendship formation and social support network measures) per study site. Cross tabulations were used to compare mechanisms for getting to know people in the neighborhood and the type of social support provided to pet owners per site. The reference categories were non-pet owners/other pet owners.

All models were adjusted for the key socio-demographic variables that are commonly taken into account in neighborhood-based studies (namely age group, sex, highest educational level, ethnicity/race, and number of children living in the household). These reflect the demographics and have been adjusted for in previous studies relating to pets [8,21,51–53].

Thematic analysis [54] was undertaken on the full verbatim text from the open-ended response question—this was initially undertaken by one member of the research team, who developed a coding frame based on the themes that emerged. Coding categories related to type of pet referred to (all types were coded so the category included common pet types such as dog or cat, as well as other infrequently mentioned types such as donkey, sheep or snake); nature of interaction (e.g. met someone didn’t know before, became friends, cooperated together as a result) and whether this comment suggested the “getting to know people” experience was positive, neutral or negative (such as animosity between neighbors as a result of a dog fight). The qualitative data and coding frame was then reviewed by the study’s lead investigator. Once there was consensus on the coding frame, two members of the research team undertook independent coding of the responses. There was agreement between coders on >95% of the responses. Where there were discrepancies, the coding was reviewed and decided by the lead investigator.

## Results

### Getting to know people in the neighborhood

Over 80% of respondents reported they had got to know people in their neighborhood whom they didn’t know previously (San Diego 78.4%; Nashville 81.5%; Portland 84.5%; and Perth 85.9%). These respondents (*n* = 2223) were then asked how this occurred. Table 1 summarizes the top five ways in which people reported getting to know people. After proximity factors (being neighbors and through local streets and park), getting to know people through pets was the next most commonly identified facilitator in three cities (San Diego, Nashville, Perth), while in Portland pets were the fourth most common facilitator. ‘Dog walking’ was specifically mentioned as a mechanism for getting to know others by 9.6% of respondents in San Diego, 13.4% in Perth, 10% in Nashville and 7.6% of respondents in Portland.

**Table 1:** Getting to know people in neighborhood and mechanisms for doing so (pet and non-pet owners).

		**Total (n = 2692)**	**San Diego (n = 690)**	**Nashville (n = 664)**	**Portland (n = 634)**	**Perth (n = 704)**
		**n (%)**	**n (%)**	**n (%)**	**n (%)**	**n (%)**
**Got to know people in neighborhood whom didn’t know previously**		2223 (82.6)	541 (78.4)	541 (81.5)	536 (84.5)	605 (85.9)
Pet owner		1362 (86.3)	315 (83.3)	335 (86.3)	330 (85.9)	382 (89.0)
Non-pet owner		861 (77.4)	226 (72.4%)	206 (74.6)	206 (82.4)	223 (81.1)
**How got to know these people** ^*+*^						
Being neighbors		1720 (63.9)	410 (75.8)	422 (78.0)	413 (77.1)	475 (78.5)
Children’s school		217 (8.1)	51 (9.4)	36 (6.7)	50 (9.3)	80 (13.2)
Local street or park		412 (15.3)	89 (16.5)	90 (16.6)	74 (13.8)	159 (26.3)
Community event		231 (8.6)	47 (8.7)	55 (10.2)	56 (10.4)	73 (12.1)
Through pets:	Generally	297 (11.0)	67 (12.4)	72 (13.3)	55 (10.3)	103 (17.0)
	Walking my/a dog	228 (8.5)	52 (9.6)	54 (10.0)	41 (7.6)	81 (13.4)
	Other than dog walking	106 (3.9)	28 (5.2)	31 (5.7)	18 (3.4)	29 (4.8)

*Totals for columns > 100% as more than one response was accepted*.

*+ top five responses*

*56.*0% of dog owners walk their dog*

In the ordinal regression, pet owners were significantly more likely to get to know people in their neighborhood whom they didn’t know previously, compared with non-pet owners (OR1.61, 95% CI: 1.30, 1.99). This pattern was observed in all four cities, but did not reach statistical significance for Portland (San Diego: OR 1.85, 95%CI 1.30, 1.99; Nashville: OR 1.91, 95%CI 1.25, 2.74; Portland: OR 1.12, 95%CI 0.71, 1.79; Perth: OR 1.76, 95%CI 1.11, 2.80).

### Pet ownership

Overall, 58.7% of respondents owned a pet, with some small variation between the four cities (San Diego 54.8%, Nashville 58.4%, Portland 60.6% and Perth 60.9%). Dogs were the most commonly owned pet in all four cities (see Table 2 for details), followed by cats, fish and birds. If respondents reported owning a pet other than a dog, cat, fish or bird, they were asked what type of pet this was. Examples included chickens, rabbits, hamsters, guinea pigs, turtles, tortoises and lizards.

**Table 2:** Types of social relatedness facilitated through pets among Pet Owners.

	**San Diego (n = 378)**		**Nashville (n = 388)**		**Portland (n = 384)**		**Perth (n = 429)**	
	**n**	**%**	**n**	**%**	**n**	**%**	**n**	**%**
**Got to know people in neighborhood as a result of pet**	193	51.1	204	52.6	183	47.7	229	53.4
**Regard at least one person met through pet as a friend**	102	27.0	123	31.7	89	23.2	106	24.7
**Received at least one form of social support from people met through pet**	155	41.0	180	46.4	149	38.8	184	42.9
**Type of social support provided by person/people met through pet**								
Informational	121	32.0	149	38.4	116	30.2	168	39.2
Instrumental	128	33.9	154	39.7	122	31.8	132	30.8
Appraisal	100	26.5	116	29.9	98	25.5	118	27.5
Emotional	68	18.0	79	20.4	53	13.8	79	18.4

### Getting to know people in the neighborhood through pets

Among pet owners, the proportions reporting they got to know people in the neighborhood as a direct result of their pet were similar across the four cities (San Diego 51.1%; Nashville 52.6%; Portland 47.7%; and Perth 53.4%) (See Table 2).

In each of the four cities, dog owners were more likely than other pet owners to report they had got to know people in the neighborhood through their pet (see Table 3 for odds ratios for overall sample and by city). Specifically, dog owners were five times more likely to get to know people in their neighborhood than other pet owners in the overall sample (OR 5.01, 95%CI: 3.90, 6.43). In the three U.S. cities, the odds of dog owners having got to know people in the neighborhood through their pet was between five to six times more likely, compared with other pet owners, and over four times more likely in Perth.

**Table 3:** Odds ratios (95% CI) for getting to know people, friendship formation and social support by dog compared with other pet ownership.

	**Type of pet**	**Overall (n = 1597)**	**San Diego**	**Nashville**	**Portland**	**Perth**
**Got to know people in the neighborhood as a direct result of their pet**	Dog Owner	**5.01 (3.90, 6.43)** **	**6.76 (3.79,12.06)** **	**5.81 (3.24, 10.4)** **	**5.15 (3.20, 8.31)** **	**4.37 (2.70, 7.06)** **
	Other Pet Owner^	1.00	1.00	1.00	1.00	1.00
**Regard at least one person met through pet as a friend**	Dog Owner	**2.59 (1.94, 3.46)** **	**3.64 (1.88, 7.05)** **	**3.28 (1.74, 6.18)** **	**2.64 (1.49 4.66)** *	1.69 (0.98, 2.90)
	Other Pet Owner^	1.00	1.00	1.00	1.00	1.00
**Received at least one form of social support from people met through pet**	Dog Owner	**3.93 (3.04, 5.09)** **	**4.55 (2.55, 8.12)** **	**4.14 (2.34, 7.32)** **	**4.77 (2.88, 7.90)** **	**3.20 (1.96, 5.20)** **
	Other Pet Owner^	1.00	1.00	1.00	1.00	1.00

^Adjusted for age group, sex, highest education level completed, ethnicity/race and number of children living in household. Reference group: other pet owner.

*P < 0.05

** *P* < 0.001

When dog owners were stratified by whether they walked their dog or not, those who walked their dog were significantly more likely to get to know people through their pet than dog owners who didn’t walk their dog (OR 3.10, 95%CI 2.33–4.12). This was significant (p<0.001) for all four cities (San Diego: OR 4.83, 95%CI 2.56–9.11; Nashville: OR 3.91, 95%CI 2.08–7.37; Portland: OR 2.86, 95%CI 1.64–4.97; Perth: OR 2.60, 95%CI 1.46–4.64) (data not shown in Table).

Those who had met people through their pet were asked if they could elaborate on how this occurred, and 789 responses (560 in the U.S. cities, 229 in Perth) were received for this open-ended question. These open-ended comments helped illustrate a variety of ways pets had precipitated people getting to know others in their neighborhood, with the role of pets as social ‘ice-breakers’ a recurring theme. As reflected in the quotes below, this role of pets as an ice-breaker is not confined to exchanges with other pet owners, but can extend also to the neighborhood populace more generally:

*“People always stop*, *complete strangers will stop*, *and talk to you about your dog and ask you about it*. *It's funny that it seems to be an ice-breaker*, *or maybe people with dogs are that particular way” (male*, *Perth)*.

*“I tend to talk to people who I wouldn't normally talk to*. *Without the dog*, *I wouldn't speak to them” (male*, *Portland)*.

*“Back in the day when we used to have cats the cat used to sit on the top of the step outside and people would say hi because of the cat*. *Now we have two dogs and when we were walking our dogs there was actually a person looking for a play date for their dog so we got to know people like that” (female*, *Nashville)*.

Although many of the responses received related to dogs, other types of pets were also mentioned most often relating to cats, but there were also examples about other animals kept as pets, including chickens, rabbits, sheep, turtles, a donkey and a pet snake:

*“When we were first moving into the neighborhood some neighbors came over and noticed our cats and said if you need someone to watch your cats while you are away*, *we’ll be happy to do it*. *Another neighbor has a couple of cats as well and we initially got to know one another as we were watching the cats work things out among themselves*. *It made it easy to get to know the neighbors in an otherwise established neighborhood*. *That interaction made us feel welcome” (male*, *Portland)*.

*“Their children are interested in seeing the snake and we never let children come in without parent permission*. *So before anyone can see the snake or handle the snake we need to have met the parents and had it okayed with them” (female*, *Perth)*.

In the empirical analysis, dog owners were more likely to report getting to know people through their pet than non-dog owners, and many of the open-ended responses illustrated the way dogs or dog walking facilitates social interaction:

*“Lots of folks in this neighborhood own and walk dogs*. *The dogs insist on meeting and greeting*, *and their humans follow suit*. *It has caused me to be more social than is my inclination” (male*, *Portland)*.

*“People walking their dog*, *and stopping to have a chat*. *Dogs and owners communicate with each other” (male*, *Perth)*.

*“When we first got our dog Toby*, *as a puppy we had him out front playing and we had several neighbors come over to meet him and play with him*. *I got to meet neighbors that I had not met before” (female*, *Nashville)*.

A number of quotes alluded to a sense of ‘something in common’ experienced with other pet owners, particularly if of the same species:

*“I was just visiting with one of them and we mentioned that we had a rabbit and they had a rabbit too*. *They became more than just acquaintances” (female*, *Portland)*.

*“I like meeting my pet owner neighbors*. *I feel like I have something in common with them” (male*, *Nashville)*.

*“Had dog in front yard*, *new neighbor admired her and commented that they had one like her growing up and they missed it*. *We have remained friends” (male*, *San Diego)*.

Less than 3% of the open-ended responses gave any indication of a negative experience of meeting other people as a result of their pet. The negative experiences pertained mostly to either a neighbor complaining about dog barking, or dog or cat fights, but in one of the cat fight examples, the respondent went on to indicate that a friendship was forged with the other cat owner as a consequence.

### Friendship formation facilitated through pets

In each of the four cities, around a quarter of pet owners who got to know people in the neighborhood through their pet considered one or more of the people they met to be friends (i.e., more than just an acquaintance) (San Diego 27.0%; Nashville 23.2%; Portland 31.7%; and Perth 24.7%) (See Table 2).

Having pets as a point ‘in common’ was one of the observed precipitants of friendship formation:

*“It's made me think that we have a great deal in common*. *We found that we are like minded about some other things*. *Having our cats as a point in common has made it easier for us to become friends” (female*, *Nashville)*.

*“Whenever I head out for my walks out to the park with my dog*, *I bump into the same people who also walk their dog and through this you become friends with the people” (male*, *Perth)*.

The propensity for pets to bring people into contact with neighbors in unanticipated ways was also noted as a trigger for friendship formation:

*“When we first got our dog Vegas from a neighbor and every time we opened the door he would race out and go to the neighbor’s house*, *so every time we had to chase him*, *we got to meet more neighbors*. *People who have lost their pets go around the neighborhood and ask if we have seen this animal*, *and we all go out looking for it and then we go out and have coffee afterwards” (female*, *Portland)*.

*“The cat steals people’s socks from their houses*, *and then I return them*. *It's a good way to get to know people*. *They all think it is hilarious” (female*, *Perth)*.

In the following examples, dog owners describe how their dog had precipitated the formation of new friendships:

*“I've meet 3 neighbors while we were walking our dogs at the nearby park*. *Through the dogs we have met some good people*, *new friends” (male*, *Portland)*.

*“There is a path in our neighborhood that people walk along with their dogs*. *When you walk that path at the same time every day you run into the same people and start conversations and make friends” (female*, *San Diego)*.

### Social support received by pet owners from people met through their pet/s

Overall, 42.3% of pet owners received one or more types of social support (see Table 2) from people they reported getting to know through their pet. Compared with other pet owners, dog owners were more than three times more likely to receive at least one type of social support from people met through their pet (OR 3.93, 95%CI 3.04, 5.09). Table 3 provides the odds ratios for each city comparing social support between dog and other pet owners.

Some minor differences were observed when the U.S. cities were compared with the Australian city with respect to the four types of social support most commonly received. For example, in the U.S. cities, instrumental support (e.g. borrows something, practical help, feed a pet or collect mail while away) was the most common, with around one third of pet owners in each city indicating that, through their pet, they had met someone they felt they could ask for help in some practical way. Informational support (e.g. provision of information about local services) was the second most common in each U.S. city. In the Perth sample, informational support was the most common (39.2%) followed by instrumental support (30.8%). In all four cities, around one quarter of pet owners had met someone through their pet that they could ask for advice (appraisal support) whilst for emotional support, the proportion of pet owners reported they had met people through their pet who they could talk with about something that was worrying them ranged from 13.8% (Portland) to 20.4% (Nashville).

## Discussion

Previously described as a bridge between humans and nature [55], the results of this study suggest companion animals can also act as a social bridge between people and human inter-relatedness in a number of ways. HAI research to date has often focused on the animals themselves as a source of companionship and social support for their human companions [32,33]. This study used an alternative lens to investigate the role of pets as a conduit for several forms of human social relatedness; getting to know other people, friendship formation and social support. Like many other social mammals, humans are a relational species, but the isolating and fast-paced nature of modern living tends to minimize the capacity for human-to-human contact [56]. While many pet owners have anecdotes about social interactions that occur with other people when they are in the company of their pet, whether this evolves into friendships or socially supportive networks has rarely been investigated empirically.

Compared with non-pet owners, pet owners were more likely to have got to know people in their suburb that they didn’t previously know. This difference was significant in three cities (San Diego, Nashville and Perth) and is congruent with the findings of an earlier Perth study [8]. In an unprompted question about how people got to know others in their neighborhood, pets emerged consistently in the ‘top five’ factors in all four cities, with dog walking the fourth most frequently mentioned way in which people got to know others in their neighborhood in San Diego and Perth, fifth in Nashville and sixth in Portland. This is a pertinent finding, given mounting public lament and social policy discourse about the socially isolating nature of modern suburban living [19,57]. Increasing car dependence, traffic density, time-poor life styles, diminishing front yards, fewer porches and drive-in garages are among trends that have eroded some incidental opportunities for contact with neighbors [19]. Community facilities and events are sometimes lauded as ways to counter this, but interestingly, in this study, dog walking and pets were more frequently reported as facilitators of getting to know people than either of these.

So how and why do pets facilitate people getting to know one another? This study supports observational research indicating the role of animals as ‘ice-breakers’ and catalysts for social interaction or conversation between strangers [23–25,31]. This is notable given strangers are more commonly observed to politely disregard each other in public settings [58,59]. Newby [20p180] purports *“the presence of a pet seems to ‘normalize’ social situations*, *getting everyone through the ice-breaker stage to the point where they can risk directly engaging with the unfamiliar person*.” Such incidental and informal social interaction is important, as it is psychologically beneficial to feel some sense of connection with those amongst whom we live [29].

Previous research has commented on the role of dog walking as a conduit for social opportunities to meet others and converse [3,23,26]. In this study we had sufficient sample size to explore differences between dog owners and other pet owners, and between those who walk their dog and those who do not. Given dogs are the most likely pet type to draw owners out of their homes and into the broader community, it was not surprising to find dog owners were significantly more likely to have met people or made friends through their pet compared with owners of other types of pets. Across the four cities, dog owners were five times more likely to have got to know people in their neighborhood, and more than twice as likely to report having made friends through their pet.

Both the empirical data and open-ended responses in this study indicated other types of pets as well as dogs can act as a social lubricant. As with dogs, other pets also appear to be a potential conversational ‘ice-breaker,’ with many respondents providing examples of pets other than dogs serving as an initial conversation starter. In some of the other examples provided, pets had precipitated an interaction between strangers that then evolved into a social relationship or friendship. Secondly, pet owners (regardless of the type of pet), seem to find an affinity with other pet owners; they connected through a shared love of animals [20], with the exchange of pet anecdotes a common ‘ice-breaker’ [22].

Beyond incidental social contact and more generalized notions of getting to know people, the study sought to investigate more specifically whether pet-facilitated social contacts translate into tangible friendships or sources of social support. Our findings affirmed this premise, countering an earlier UK study which concluded casual interactions facilitated by dogs do not necessarily enhance social networks or social support [34]. Specifically, we found that across the four cities, around a quarter of pet owners who got to know people in the neighborhood through their pet considered one or more of the people they met to be friends, and 42% of pet owners had received one or more forms of social support from people met through their pet. It is pertinent to note the wording of the question asked about *actual* support received, and was not just about the perceived or hypothetical availability of social support.

The findings relating to pets as one of the potential conduits for socially supportive networks have significant currency given the strong and still growing evidence for the importance of social support and social relationships for a prism of health and wellbeing outcomes [60] ranging from lower rates of morbidity and mortality, stress reduction and mental health [5,61]. Although this study asked people about the *actual* receipt of social support from others, it has been compellingly argued elsewhere that even knowing that socially supportive networks exist should the need arise, is psychologically beneficial [62].

While social support is often referred to and measured as a unidimensional concept, in this study we were particularly interested in disaggregating social support into the four different forms it might take [46,47]. This ranged from favors and practical help (*instrumental support*); through to the provision of *informational support*; advice or opinions (*appraisal support*); or emotional *support* such as empathy or encouragement. Similar patterns of variability across the four types of social support were observed in all four cities, with emotional support the least common, although still received by 17% of pet owners from people met through their pet. This makes intuitive sense, as listening to someone else’s problems or providing affection requires more personal investment than favors of a more practical or information-giving nature.

The disaggregation of different forms of social support derived from people met through pets is a novel contribution to the HAI literature, as it testifies to the merits of more fine-tuned investigation of the social exchanges and relationships often collectively badged as ‘social lubrication’. Unfortunately, limitations with the data collected in this study did not enable us to explore further nuances, such as whether the people who provide emotional support or advice are the same as those who provide practical help or favors. Social network analysis is one methodology that could be used to elucidate this in future studies.

### Study strengths and Limitations

The large sample size of this study is a strength, as much of the research in the HAI field has been hindered by methodological limitations such as small sample sizes [63]. Some of the items had been previously used in a smaller study in Perth, Australia [8] and yielded reasonably congruent results in this larger multi-city study, suggesting that earlier findings were not a one-off phenomenon. It is acknowledged, however, that the four cities in this study cannot be construed to be representative of the two countries. Items would need to be included in some form of nationally representative population survey to enable conclusions to be extrapolated to the country comparison level.

Moreover, there were some differences in results observed between cities, and explanatory hypotheses for this could be better explored if there were also objective measures of variability in housing type, pet related laws and regulations, neighborhood walkability and urban form, park and green space access, and safety. There may also be socio-cultural differences between cities that influence the propensity of residents to welcome contact with people they don’t know or turn to others for social support.

The investigators sought to standardize the season of survey administration between the two countries (autumn/early winter), as it is possible findings within a city will vary according to season. For instance, summer is typically the season most conducive to walking a dog or lingering outdoors engaging in social interaction with neighbors, hence this may have had some influence on saliency and recall. The findings may also be less able to be generalized to cities where the climate is less conducive to people walking, spending time outdoors (with or without pets) or ‘getting out of the house’ and interacting with others in their local area. Anecdotal evidence from cold climate cities such as Edmonton, Canada, however, suggests dogs in particular are often a motivator for their owners to go out walking regardless of weather [64].

The response rate of 47% can be considered reasonable-to-good for a telephone survey of similar completion time (in this case about 25 minutes) [65] and was greater than the response rate of 34.3% obtained in the previously published study on social capital and pet ownership [8]. Nonetheless, we acknowledge non-response bias is a limitation of the study. That the gender, age and pet ownership rates of the sample were similar to the general population in each country suggests the sample of responders was reasonably representative of the populations.

As with all cross-sectional study designs, causality cannot be established. We also cannot discount there may be personality or temperament traits that have a bearing on neighborhood perceptions, sociability and pet ownership. The use of self-report data and thus recall bias is also acknowledged.

We were unable to investigate whether the number of years lived within in a neighborhood had a bearing on the likelihood of relationships through pets being formed. Additionally, data regarding the geographic proximity of people with whom pet owners became friends or received social support as a result of their pet, hence we could not ascertain to what extent proximity might influence the nature of relationships that develop. The addition of geo-coded address data or social network analysis with proximity measures included would be useful angles for future research.

Finally, we are aware not all social interactions or relationships facilitated by pets are necessarily positive, and, as demonstrated in the social capital literature [66,67], there can be a downside to some forms of social connectedness. The scope of this study did not permit this area to be explored, but there would be merit in including this in future studies.

### Future Research

In addition to some of the suggestions for future research spawned by preceding discussion of this study’s limitations, further international collaborative research in this area is encouraged. Both the U.S. and Australia are countries with relatively high levels of pet (and particularly dog) ownership, so it would be interesting to conduct this type of research in other countries where levels or type of pet ownership may differ.

‘Pet culture’ differences may also have a bearing on some findings, so any international research would need to include consideration of the socio-cultural context of each study site. In the U.S. for example, dedicated dog runs within parks are more common than in Australia, and may foster greater social interaction between dog walkers than a regular park. There are also differences between countries and even between cities within the same country in relation to the permissibility of pets in various settings such as cafes, apartments and flats, and on public transit. The opportunity to meet or get to know people or forge social support networks through pets will in part be influenced by both the contextual laws and social norms relating to the presence of pets in such settings.

Collecting more of this type of rich contextual data would be a useful addition to future studies wishing to further explore the differences between cities. Complementary qualitative data collection (for example interviews with residents or ethnographic approaches) could also enrich our understanding of the influence of the local contextual environment or socio-cultural norms.

Further research in this area need not be confined only to studies with a primary pet or companion animal focus, as the set of items used in this paper is small enough that it could be incorporated into data collection being undertaken for another primary purpose. Indeed, inclusion of some pet related items in longitudinal neighborhood studies collecting geo-spatial data would be particularly useful, as this could potentially help answer some of the questions regarding the physical or socio-cultural characteristics of different neighborhood or city environments influence on the pattern of pet-facilitated interactions. The inclusion of some key pet items in large-scale population health surveys or even the national census could yield rich new sources of data.

## Conclusion

This is the first multinational study to concurrently explore the role of pets as a catalyst for different forms of social relatedness. Pet ownership appears to be a significant factor for facilitating social interaction and friendship formation within neighborhoods. For pet owners, this also translates into new sources of social support, both of a practical and emotionally supportive nature. Given friendship networks and social support are associated with mental health and wellbeing of communities; supporting pet ownership may well be an under-recognized conduit for individual and community wellbeing.

## Supporting Information

S1 Appendix Sample characteristics by city. (DOCX)

## References

[1] Allen K, Shykoff B, Izzo J (2001) Pet ownership, but not ace inhibitor therapy, blunts home blood pressure responses to mental stress. Hypertension 38: 815–820. 11641292

[2] Headey B, Grabka MM (2007) Pets and human health in Germany and Australia: National longitudinal results. Social Indicators Research 80: 297–311.

[3] Wells DL (2007) Domestic dogs and human health: An overview. British journal of health psychology 12: 145–156. 17288671 10.1348/135910706X103284

[4] Headey B, Na F, Zheng R (2008) Pet dogs benefit owners’ health: A ‘natural experiment’in China. Social Indicators Research 87: 481–493.

[5] Holt-Lunstad J, Smith TB, Layton JB (2010) Social relationships and mortality risk: a meta-analytic review. PLoS Medicine 7: e1000316 10.1371/journal.pmed.1000316 20668659 PMC2910600

[6] Shrapnel B (2006) Contribution of the pet care industry to the Australian economy Australian Companion Animal Council.

[7] APPMA (2011) 2011/2012 National Pet Owners Survey. Greenwich: American Pet Products Manufacturers Association.

[8] Wood L, Giles-Corti B, Bulsara M (2005) The pet connection: pets as a conduit for social capital?. Social Science and Medicine 61: 1159–1173. 15970228 10.1016/j.socscimed.2005.01.017

[9] Beck A, Meyers N (1996) Health enhancement and companion animal ownership. Annual Review of Public Health 17: 247–257. 8724226 10.1146/annurev.pu.17.050196.001335

[10] Allen K, Blascovich J (2002) Anger and Hostility Among Married Couple: Pet Dogs as Moderators of Cardiovascular Reactivity to Stress. Delta Society.

[11] Qureshi AI, Memon MZ, Vazquez G, Suri MFK (2009) Cat ownership and the Risk of Fatal Cardiovascular Diseases. Results from the Second National Health and Nutrition Examination Study Mortality Follow-up Study. Journal of vascular and interventional neurology 2: 132 22518240 PMC3317329

[12] Edney A (1995) Companion animals and human health: an overview. [see comments]. Journal of the Royal Society of Medicine 88: 704p–708p. 8786595 10.1177/014107689508801220 PMC1295422

[13] Friedmann E, Thomas S (1995) Pet ownership, social support, and one-year survival after acute myocardial infarction in the Cardiac Arrhythmia Suppression Trial (CAST). American Journal of Cardiology 76: 1213–1217. 7502998 10.1016/s0002-9149(99)80343-9

[14] Cutt H, Knuiman M, Giles-Corti B (2008) Does getting a dog increase recreational walking? Int J Behav Nut Phys Act 5. 10.1186/1479-5868-5-17 PMC2359767 18366804

[15] Jofre M (2005) [Animal-assisted therapy in health care facilities]. Revista chilena de infectologia: organo oficial de la Sociedad Chilena de Infectologia 22: 257–263. 16077894 10.4067/s0716-10182005000300007

[16] Arambašić L, Keresteš G, Kuterovac-Jagodić G, Vizek-Vidović V (2000) The role of pet ownership as a possible buffer variable in traumatic experiences. Studia Psychologica 42: 135–146.

[17] Mulcahy C, McLaughlin D (2013) Is the Tail Wagging the Dog? A Review of the Evidence for Prison Animal Programs. Australian Psychologist.

[18] Shankar A, McMunn A, Banks J, Steptoe A (2011) Loneliness, social isolation, and behavioral and biological health indicators in older adults. Health Psychology 30: 377–385. 10.1037/a0022826 21534675

[19] Leigh A (2011) Disconnected: NewSouth Publishing.

[20] Newby J (1997) The Pact for Survival: Humans and their Companions Sydney: ABC Books. 280 p.

[21] Wood L, Giles-Corti B, Bulsara MK, Bosch D (2007) More than a furry companion: animals on neighbourhood interactions and sense of community. Society and Animals 15: 43–56.

[22] Wood L, Christian H (2011) Dog walking as a catalyst for strengthening the social fabric of the community In: Johnson R, Beck A, MCCune S, editors. The Health Benefits of Dog Walking for People and Pets Evidence & Case Studies: Purdue University Press.

[23] Messent P (1983) Social facilitation of contact with other people by pet dogs In: Katcher A, Beck A, editors. New Perspectives on Our Lives With Companion Animals. Philadelphia: University of Pennsylvania Press pp. 37–46.

[24] Robins D, Sanders C, Cahill S (1991) Dogs and their people: pet-facilitated interaction in a public setting. Journal of Contemporary Ethnography 20: 3–25.

[25] Rogers J, Hart LA, Boltz RP (1993) The role of pet dogs in casual conversations of elderly adults. The Journal of Social Psychology 133: 265–277. 8412041 10.1080/00224545.1993.9712145

[26] McNicholas J, Collis G (2000) Dogs as catalysts for social interactions: robustness of the effect. British Journal of Psychology 91 (Pt 1): 61–70. 10717771 10.1348/000712600161673

[27] Hunt S, Hart L, Gomulkiewicz R (1992) Role of small animals in social interactions between strangers. Journal of Social Psychology 132: 245–256.

[28] Mackay H (2013) What Makes Us Tick?: The ten desires that drive us Sydney Hachette Australia.

[29] Albery N (2001) The World's Greatest Ideas: An Encyclopedia of Social Inventions New Society Publishers

[30] Cutt H, Giles-Corti B, Wood L, Knuiman M, Burke V (2008) Barriers and motivators for owners walking their dog: results from qualitative research. Health Promotion Journal of Australia 19: 38–44. 10.1071/he08118 18647125

[31] Edwards V, Knight S (2006) Understanding the Psychology of Walkers with Dogs: new approaches to better management Hampshire: University of Portsmouth.

[32] Garrity T, Stallones L (1998) Effects of pet contact on human well-being In: Wilson CC, Turner DC, editors. Companion Animals in Human Health. Thousand Oaks, California: SAGE Publications, Inc. pp. 3–22.

[33] McNicholas J, Collis G (2006) Animals as Social Supports: Insights for Understanding Animal-Assisted Therapy. In: Fine AH, editor. Handbook on Animal-Assisted Therapy: Theoretical Foundations and Guidelines for Practice. pp. 49–71.

[34] Collis G, McNicholas J, Harker R (2003) Could enhanced social networks explain the association between pet ownership and health? unpublished paper, Department of Psychology, University of Warwick.

[35] e.Rebublic (2015) Population Density for U.S. Cities Map.

[36] Current Results Nexus (2015) Average Annual Temperatures for Large US Cities.

[37] United States Census Bureau (2010) American Fact Finder.

[38] Australian Bureau of Statistics (2010) National Regional Profile: Perth (C) (Local Government Area)

[39] Petcare (2015) what is "pets in the city"?

[40] Wood L, Giles-Corti B, Bulsara M (2012) Streets apart—does social capital vary with neighbourhood design? Urban Studies Research.

[41] Animal Health Alliance (2013) Pet Ownership in Australia 2013—summary.

[42] American Veterinary Association (2012) The 2012 U.S. Pet Ownership and Demographics Sourcebook

[43] Wood L (2006) Social capital, mental health and the environments in which people live (PhD thesis). Perth: The University of Western Australia.

[44] McHarg M, Baldock C, Heady B, Robinson A (1995) National People and Pets Survey: Health Benefits. Sydney: Urban Animal Management Coalition. 19–29 p.

[45] Cutt H, Giles-Corti B, Knuiman M, Pikora T (2008) Physical activity behaviour of dog owners: Development and reliability of the Dogs and Physical Activity (DAPA) tool. J Phys Act Health 5: S73–S89. 18364529 10.1123/jpah.5.s1.s73

[46] House J (1981) Work stress and social support 1981 RMA-WC, editor. Reading MA: Addison-Wesley Company.

[47] Israel BA (1985) Social networks and social support: implications for natural helper and community level interventions. Health Education & Behavior 12: 65–80. 10.1177/109019818501200106 3980242

[48] Young R (2009) Using social network analysis to explore the role of playgroups as a channel for social support, UWA Health Science Honours Project School of Population Health, The University of Western Australia.

[49] United States Census Bureau (2013) American Fact Finder.

[50] Australian Bureau of Statistics (2014) Australian Bureau of Statistics.

[51] Cutt H, Giles-Corti B, Knuiman M, Timperio A, Bull F (2008) Understanding Dog Owners' Increased Levels of Physical Activity: Results From RESIDE. Am J Public Health 98: 66–69. 18048786 10.2105/AJPH.2006.103499 PMC2156050

[52] Cutt H, Giles-Corti B, Knuiman M (2008) Encouraging physical activity through dog walking: Why don't some owners walk with their dog? Prev Med 46: 120–126. 17942146 10.1016/j.ypmed.2007.08.015

[53] Christian nee Cutt H, Giles-Corti B, Knuiman M (2010) "I'm Just a'-Walking the Dog" correlates of regular dog walking. Fam Community Health 33: 44–52. 10.1097/FCH.0b013e3181c4e208 20010004

[54] Patton M (2002) Qualitative evaluation and research methods Newbury Park, California: Sage Publications.

[55] Podberscek AL (2000) The Relationships Between People And Pets Cambridge: Cambridge University Press.

[56] Walljasper J (2007) The Great Neighborhood Book: New Society.

[57] Putnam RD (2004) Better together: Restoring the American community: SimonandSchuster. com.

[58] Patterson ML, Webb A (2002) Passing encounters: Patterns of recognition and avoidance in pedestrians. Basic and applied social psychology 24: 57.

[59] Hirschauer S (2005) On Doing Being a Stranger: The Practical Constitution of Civil Inattention. Journal for the theory of social behaviour 35: 41–67.

[60] Uchino B (2006) Social Support and Health: A Review of Physiological Processes Potentially Underlying Links to Disease Outcomes. Journal of Behavioral Medicine 29: 377–387. 16758315 10.1007/s10865-006-9056-5

[61] Thoits PA (2011) Mechanisms Linking Social Ties and Support to Physical and Mental Health. Journal of Health and Social Behavior 52: 145–161. 10.1177/0022146510395592 21673143

[62] Cohen S, Wills TA (1985) Stress, Social Support, and the Buffering Hypothesis. Psychological Bulletin 98: 310–357. 3901065

[63] Kazdin AE (2011) Single-case research designs: Methods for clinical and applied settings: Oxford University Press.

[64] Knight S, Edwards V (2008) In the Company of Wolves The Physical, Social, and Psychological Benefits of Dog Ownership. Journal of Aging and Health 20: 437–455. 10.1177/0898264308315875 18448686

[65] Brownson RC, Chang JJ, Eyler AA, Ainsworth BE, Kirtland KA, Saelens BE, et al (2004) Measuring the environment for friendliness toward physical activity: a comparison of the reliability of 3 questions. American Journal of Public Health 94: 473 14998817 10.2105/ajph.94.3.473 PMC1448279

[66] Caughy M, Campo P, Muntaner C (2003) When being alone might be better: neighbourhood poverty, social capital and child mental health. Social Science and Medicine 57: 227–237. 12765704 10.1016/s0277-9536(02)00342-8

[67] Portes A, Landolt P (1996) The Downside of Social Capital. The American Prospect 26: 18–21 (cont. to p94).

