# Supplementary material for: The Pet Factor - Companion Animals as a Conduit for Getting to Know People, Friendship Formation and Social Support
Source: PLoS One. 2015 Apr 29;10(4):e0122085. doi: 10.1371/journal.pone.0122085 (PMC4414420; doi:10.1371/journal.pone.0122085)
Supplement: S1 Appendix — (DOCX) [file pone.0122085.s001.docx]

S1 Appendix. Sample characteristics by city

|  |  | **San Diego** (n=690) | | **Nashville** (n=664) | | **Portland** (n=634) | | **Perth** (n=704) | |
| --- | --- | --- | --- | --- | --- | --- | --- | --- | --- |
|  |  | **n** | **%** | **n** | **%** | **n** | **%** | **n** | **%** |
| **Age group^1^** | 18-29 years | 165 | 23.9 | 87 | 13.1 | 62 | 9.8 | 87 | 12.4 |
|  | 30-39 years | 146 | 21.2 | 138 | 20.8 | 137 | 21.6 | 65 | 9.2 |
|  | 40-49 years | 111 | 16.1 | 143 | 21.5 | 142 | 22.4 | 106 | 15.1 |
|  | 50-59 years | 109 | 15.8 | 120 | 18.1 | 121 | 19.1 | 174 | 24.7 |
|  | 60+ years | 152 | 22.0 | 164 | 24.7 | 158 | 24.9 | 268 | 38.1 |
| **Sex** | Male | 353 | 51.2 | 287 | 43.2 | 287 | 45.3 | 345 | 49.0 |
|  | Female | 337 | 48.8 | 377 | 56.8 | 347 | 54.7 | 359 | 51.0 |
| **Highest education level^2^** | Secondary school or less | 195 | 28.3 | 207 | 31.2 | 169 | 26.7 | 288 | 40.9 |
|  | Vocational training | 178 | 25.8 | 137 | 20.6 | 149 | 23.5 | 192 | 27.3 |
|  | Bachelor degree or higher | 285 | 41.3 | 292 | 44.0 | 286 | 45.1 | 212 | 30.1 |
|  | Other | 13 | 1.9 | 15 | 2.3 | 16 | 2.5 | 6 | 0.9 |
| **Ethnicity/race** | White/Caucasian | 381 | 55.2 | 486 | 73.2 | 550 | 86.8 | ^b^ |  |
|  | Hispanic or Latino | 168 | 24.3 | 16 | 2.4 | 19 | 3.0 | ^b^ |  |
|  | Black/African American | 60 | 8.7 | 126 | 19.0 | 14 | 2.2 | ^b^ |  |
|  | Asian | 34 | 4.9 | 14 | 2.1 | 19 | 3.0 | 15 | 2.1 |
|  | Other | 36 | 5.2 | 15 | 2.3 | 19 | 3.0 | 32 | 4.5 |
|  | Australian | ^a^ |  | ^a^ |  | ^a^ |  | 657 | 93.3 |
| **Number of children in household^3^** | None | 438 | 63.5 | 439 | 66.1 | 411 | 64.8 | 504 | 71.6 |
|  | One | 100 | 14.5 | 102 | 15.4 | 79 | 12.5 | 73 | 10.4 |
|  | Two | 82 | 11.9 | 73 | 11.0 | 103 | 16.2 | 96 | 13.6 |
|  | Three or more | 63 | 9.1 | 44 | 6.6 | 31 | 4.9 | 29 | 4.1 |
| **Pet ownership** | Pet owner | 378 | 54.8 | 388 | 58.4 | 384 | 60.6 | 429 | 60.9 |
|  | Non-pet owner | 312 | 45.2 | 276 | 41.6 | 250 | 39.4 | 275 | 39.1 |

Missing data: ^1^37; ^2^52; ^3^25

^a^Not included as response option in U.S. Survey; ^b^Not included as response option in Perth Survey; missing excluded thus % do not equal 100
